# Health literacy and gender differences in colorectal cancer screening

**DOI:** 10.1093/eurpub/ckac130.158

**Published:** 2022-10-25

**Authors:** P Zanobini, C Lorini, M Giusti, V Minardi, V Possenti, M Masocco, G Mereu, R Cecconi, G Bonaccorsi

**Affiliations:** 1 Department of Health Sciences, University of Florence, Florence, Italy; 2 Department of Experimental and Clinic Medicine, University of Florence, Florence, Italy; 3 Centro Nazionale per la Prevenzione delle Mal, Istituto Superiore di Sanità, Rome, Italy; 4 Department of Prevention, Azienda Unità Sanitaria Locale Toscana Centro, Florence, Italy

## Abstract

**Introduction:**

Colorectal cancer (CRC) is one of the major causes of death worldwide. Previous research reported differences in screening adherence both by gender and socioeconomic determinants. However, little is known about the extent to which Health Literacy (HL) may affect gender differences in CRC screening rates. Here, we aimed to assess HL levels in both genders and their relations with CRC screening adherence.

**Methods:**

The study was performed within the Tuscan population sample selected in the Italian Behavioral Risk Factor Surveillance System (PASSI - Progress by local health units toward a healthier Italy) in 2017-2019. Socioeconomic status was measured by education level, occupation, financial status, and nationality, while HL by the Italian version of the 6-items European Health Literacy Survey Questionnaire (HLS-EU-Q6). Multivariate analysis was performed to investigate associations between CRC screening rates, social determinants, and HL.

**Results:**

Among 4,268 people aged 50-69 years included in PASSI, 64% undergo to CRC screening in the 2 years preceding the interview. No statistically significant differences in screening adherence were found by gender. In the multivariate analysis, the odds of adherence to CRC screening increased in both genders for being aged 60-69 years (Males: OR 1.43, 95% CI 1.12-1.82; Females: OR 1.72, 95% CI 1.37-2.14) and high education level (Males: OR 1.34, 95% CI 1.08-1.66; Females: OR 1.30, 95% CI 1.05-1.60). Males with a poor financial status and females with a low HL level were less likely to undergo CRC screening (OR 0.71, 95% CI 0.57-0.88 and OR 0.68 95% CI 0.49-0.95 respectively).

**Conclusions:**

Our findings suggest that adherence to CRC screening is associated with HL in females only, while it depends on financial status in males. Therefore, gender specific interventions, tailored on different factors, are needed to increase the CRC screening rates.

**Key messages:**

- The association between Health Literacy and colorectal cancer screening adherence differs according to gender.
- Gender specific interventions are needed to increase the colorectal cancer screening rates.

